# Comparison of Genomic Selection Models to Predict Flowering Time and Spike Grain Number in Two Hexaploid Wheat Doubled Haploid Populations

**DOI:** 10.1534/g3.115.019745

**Published:** 2015-07-22

**Authors:** Saravanan Thavamanikumar, Rudy Dolferus, Bala R. Thumma

**Affiliations:** CSIRO Agriculture, Canberra ACT 2601, Australia

**Keywords:** genomic prediction, SNP, biparental populations, genomic selection, wheat, GenPred, shared data resource

## Abstract

Genomic selection (GS) is becoming an important selection tool in crop breeding. In this study, we compared the ability of different GS models to predict time to young microspore (TYM), a flowering time-related trait, spike grain number under control conditions (SGNC) and spike grain number under osmotic stress conditions (SGNO) in two wheat biparental doubled haploid populations with unrelated parents. Prediction accuracies were compared using BayesB, Bayesian least absolute shrinkage and selection operator (Bayesian LASSO / BL), ridge regression best linear unbiased prediction (RR-BLUP), partial least square regression (PLS), and sparse partial least square regression (SPLS) models. Prediction accuracy was tested with 10-fold cross-validation within a population and with independent validation in which marker effects from one population were used to predict traits in the other population. High prediction accuracies were obtained for TYM (0.51–0.84), whereas moderate to low accuracies were observed for SGNC (0.10–0.42) and SGNO (0.27–0.46) using cross-validation. Prediction accuracies based on independent validation are generally lower than those based on cross-validation. BayesB and SPLS outperformed all other models in predicting TYM with both cross-validation and independent validation. Although the accuracies of all models are similar in predicting SGNC and SGNO with cross-validation, BayesB and SPLS had the highest accuracy in predicting SGNC with independent validation. In independent validation, accuracies of all the models increased by using only the QTL-linked markers. Results from this study indicate that BayesB and SPLS capture the linkage disequilibrium between markers and traits effectively leading to higher accuracies. Excluding markers from QTL studies reduces prediction accuracies.

Identification of molecular markers that can be used for predicting phenotypes is a major research area in plant and animal breeding. Traditionally, QTL mapping has been used to identify markers linked to traits. Another approach used to identify markers linked to traits is association mapping in which populations with broad diversity are used. Although these methods are useful in identifying markers linked to traits, their application in breeding programs is limited (Bernardo 2008). This is mainly because the individual marker effects are often small for predicting traits, especially quantitative traits, which are influenced by many genes. In a landmark article, Meuwissen *et al.* (2001) proposed a new method termed genomic selection (GS), which uses information from genome-wide markers to predict phenotypes. GS has been widely used by animal breeders (Hayes *et al.* 2009b). More recently, GS studies have been conducted in crop breeding as well as in forest tree populations (Crossa *et al.* 2010, 2013 Heffner *et al.* 2011; Burgueno *et al.* 2012; Ornella *et al.* 2012; Resende *et al.* 2012a,b,c). GS is currently operational in animal breeding programs in several countries (Schefers and Weigel 2012; Pryce and Daetwyler 2012). However, GS is still new to plant and tree breeding programs.

In GS, large numbers of markers randomly distributed across the genome are genotyped in small effective populations with high levels of LD. A high density of markers is required to maximize the chances of at least one marker being in LD with the QTL. Effects of all the markers are used simultaneously to develop prediction models in “training” populations (Heffner *et al.* 2009). Because the number of predictors (markers) is generally much higher than the sample size (*P* > > *n*), fixed regression methods using ordinary least squares cannot be used for developing prediction models. Statistical methods that treat marker effects as random, such as ridge regression best linear unbiased prediction (RR-BLUP) and various Bayesian models, are used for developing prediction models. The estimated model is then used to predict phenotypes in a “testing” population using only the marker genotype information. The predictions generated using marker genotypes [genomic estimated breeding values (GEBVs)] are used to select individuals without phenotypic data. The accuracy of GS is assessed by correlating GEBVs with the breeding values predicted using traditional methods that utilize phenotypic data (Heffner *et al.* 2009).

A major advantage of using GS in crop breeding is the acceleration of genetic improvement per unit-time through the reduction in time required to complete breeding cycles (Hickey *et al.* 2014). Several GS models have been developed for predicting phenotypes using large numbers of markers. These models mainly differ in the assumptions of marker effects contributing to total variance. Methods such as RR-BLUP assume that marker effects are homogeneously distributed across the loci, whereas Bayes methods allow for heterogeneity among markers, with some markers having higher effects than others (Goddard and Hayes 2007; Pérez *et al.* 2010). In methods such as BayesB, priors are used to select the number of markers with large effects; in LASSO (least absolute shrinkage and selection operator) models, penalties are used to select for the markers with major effects (Daetwyler *et al.* 2010). BayesB and Bayesian LASSO (BL) methods can identify a subset of markers with large effect (variable selection) and use them for making predictions. Reduced dimension regression methods such as principal components regression (PCR) and partial least squares (PLS) extract the latent variables for making predictions on the response (Jannink *et al.* 2010). Although PLS uses response variable of the regression for selecting latent components (supervised method), PCR does not take into account the dependent variable when selecting latent components (unsupervised method); therefore, it is not optimal for making predictions (Boulesteix and Strimmer 2007). PLS is mainly developed to deal with many correlated predictors and relatively few samples (Mevik and Wehrens 2007). Although dimension reduction with PLS is effective for dealing with the problems of small samples compared to predictors (*P* > > *n*) and multi-collinearity among the predictors, all predictors are used in the final model. Chung and Keles (2010) have proposed sparse partial least square regression (SPLS), a variant of PLS that can simultaneously reduce the dimensions and subselect the variables in prediction models.

We tested five models (BayesB, BL, RR-BLUP, PLS, and SPLS) to test prediction accuracies for time to young microspore (TYM) stage and spike grain number (SGN) in two hexaploid wheat biparental doubled haploid (DH) populations. The data used in this study were obtained from two QTL mapping experiments, where a wheat DH population from a biparental Cranbrook × Halberd (C×H) cross was phenotyped under controlled environment conditions using a hydroponics infrastructure (R. Dolferus, X. Ji, S. Thavamanikumar, E. Tanaka, J. Edlington, K. Forrest, G. Rebetzke, M. Hayden, and B. Cullis, unpublished data) and where QTL mapping for a second DH population from a Sundor × AUS30604 (S×A) cross, which was phenotyped for the same traits, is currently in progress (Dolferus *et al.*, unpublished data). The aim of the experiment was to identify QTL for maintenance of SGN under osmotic stress conditions. The young microspore stage of pollen development is the stage of reproductive development that is most sensitive to abiotic stresses (Ji *et al.* 2010). Because the lines of the DH population segregate for flowering time, plants were treated at the same young microspore stage independent of flowering time. The traits that were phenotyped for the individual lines of the DH populations were SGN under control and osmotic stress treatments (SGNC and SGNO), as well as the time for the individual plants to reach the young microspore stage of pollen development (TYM).

The main objectives of this study are to compare the accuracies of different GS prediction models and to compare the accuracies of two traits with contrasting genetic architecture using several hundreds of random markers and a few QTL-linked markers.

## Materials and Methods

### Plant materials and trait measurements

Two wheat doubled haploid (DH) populations were used in this study. This includes a population of 165 DH lines from a cross between Cranbrook and Halberd (C×H) (Chalmers *et al.* 2001) and 159 DH lines from a cross between Sundor and AUS30604 (S×A) (Dolferus, unpublished data). These two populations were subjected to osmotic stress experiments using a hydroponics facility and NaCl as osmoticum in a glasshouse to identify QTL for osmotic stress and drought resistance in two separate studies (R. Dolferus, X. Ji, S.Thavamanikumar, E. Tanaka, J. Edlington, K. Forrest, G. Rebetzke, M. Hayden, and B. Cullis, unpublished data). The date when the plants had reached a stage where the auricle distance was between −1 and +4 was recorded as TYM. From this day, the plants were stressed for 5 d with NaCl as an osmoticum using a hydroponics system (R. Dolferus, X. Ji, S. Thavamanikumar, E. Tanaka, J. Edlington, K. Forrest, G. Rebetzke, M. Hayden and B. Cullis, unpublished data). Unstressed control plants were kept in the master tank, and plants for stress treatments were treated with salt in a smaller tank in the same glasshouse. At maturity, stressed and control spikes were harvested individually and SGN (SGNO and SGNC) were determined. Two biological repeat experiments were performed between October and December in 2009 and 2010 to get independent estimates. A mixed model was fitted in ASReml 3.0 (VSN International 2009) by fitting the response variable (TYM or SGNC or SGNO) as a fixed effect and by fitting the line effects and effects due to repeated measurements as random effects. Using this model, best linear unbiased predictions (BLUP) were estimated for the three traits. BLUP for TYM, SGNC, and SGNO were used in genomic prediction models.

### SNP genotyping

DH lines from both crosses were genotyped using a 90K SNP chip developed using gene-specific SNPs, which provides a dense coverage of the wheat genome (Wang *et al.* 2014). After removing monomorphic sites, totals of 17,328 and 17,293 SNPs were obtained in C×H and S×A populations, respectively. After removing SNPs that are in complete LD (redundant markers), only 1975 and 1483 were left in C×H and S×A populations, respectively. Of these nonredundant SNPs, 808 SNPs are common to both the populations. Both sets of SNPs (population-specific and common SNPs) were used in the genomic prediction analyses. The main reason for removing these redundant markers is that they provide redundant information and will not add any power to genomic prediction analyses. Moreover, including these redundant SNPs may increase the computing time of genomic prediction analyses. Missing genotypes were imputed with mean imputation with the RR-BLUP package as well as with “rfImpute,” which uses a proximity matrix to fill-in missing values using the R package “randomForest” (Liaw and Wiener 2002).

Raw genotype data and trait (BLUPs) data are provided in supporting information, File S1, File S2, File S3, File S4, and File S5.

### Genomic prediction models

#### Prediction models:

We used different statistical models that treat marker effects as random. Five prediction models were used: RR-BLUP, BL, BayesB, PLS, and SPLS. All prediction models were tested using different packages in the R statistical environment (R Development Core Team 2011). The basic prediction model is represented as

$$y_{i}=\overset{p}{\underset{k=1}{\sum }}x_{ik}\beta_{k}+e_{i},$$

where *y_i_* is the phenotype of individual *i*, $x_{i}$ is the 1 × *p* vector of SNP genotypes of individual *i* at locus *k* of *p* loci, and *β_k_* is the effect of SNP *k*, and *e_i_* is the residual term.

The RR-BLUP model assumes homogenous variance of all markers and shrinks all marker effects equally to zero. RR-BLUP is equivalent to BLUP and uses the realized relationship matrix estimated from the markers. RR-BLUP was implemented using the “rrBLUP” package (Endelman 2011). The BL model assumes marker-specific shrinkage related to the absolute value of the estimated regression coefficient. BL models shrink markers with zero effect more than those with large effects, leading to variable selection when making predictions. BL models were implemented using “BLR” (Pérez *et al.* 2010) and “BGLR” packages (Pérez and de los campos 2014).

The BayesB model, which was proposed by Meuwissen *et al.* (2001), assumes unique variance for each marker and a proportion (π) of markers to have large effects while most of the markers have zero effect. Marker effects are estimated with Monte Carlo Markov Chain (MCMC) simulations. We used a value of 0.95 for π, and the model was run for 5000 iterations with a burn-in period of 1000 iterations. We used “BGLR” and “GenSel” packages to implement BayesB model. PLS is a dimension reduction regression that identifies the latent components that explain most of the variation in the response variable to make predictions. The optimum number of components that minimized the prediction error was selected by 10-fold cross-validation using the training samples. The optimum number of components selected from the previous step was used to predict traits in the testing population. PLS model was implemented using the “PLSR” package (Mevik and Wehrens 2007). In SPLS, in addition to the number of components, the optimum number of variables was selected based on mean squared prediction error by 10-fold cross-validation (CV) of the training samples. The selected optimum components and variables were used to predict traits in testing population. SPLS models were implemented using the “SPLS” package (Chung *et al.* 2013).

### Estimating accuracy of genomic predictions

#### Cross-validation:

Prediction accuracies of the markers within each of the two DH populations were evaluated using a 10-fold CV process (Pérez *et al.* 2010). Each population (C×H or S×A) was divided into 10 folds and individuals from the population were assigned to each fold randomly. Marker and phenotype data (BLUPs) from the nine folds were collectively used to predict the phenotypes (GEBVs) of individuals assigned to the tenth fold using only the marker data. This process was repeated 10 times. At each step, the predictive accuracy of the markers was assessed by Pearson’s correlation between the predicted values and the phenotypes. Average of the 10 left out folds was reported as accuracy of the prediction. SEs were estimated from 10 estimates of accuracies.

#### Independent validation:

In independent validation, the prediction model developed using the marker and phenotype data in one population (C×H, training population) was used to predict the phenotypes in the other population using only the marker information (S×A, testing population). Prediction accuracy of the markers was estimated by Pearson’s correlation between the GEBVs and the phenotypic data in the testing population.

### Data availability

File S1 contains genotype data for 1975 SNPs and trait data for CxH population. File S2 contains genotype data for 1483 SNPs and trait data for SxA population. File S3 contains genotype data for common 808 SNPs and trait data for both CxH and SxA population. File S4 contains genotype data for 42 SNPs from chromosome 5A and trait data for both CxH and SxA population. File S4 contains genotype data for 766 SNPs excluding 42 SNPs from chromosome 5A and trait data for both CxH and SxA population.

## Results

Estimates of broad sense heritability were generally higher for TYM (0.74 in S×A and 0.96 in C×H) and lower for SGNC (0.39 in C×H and 0.29 in S×A) and SGNO (0.40 in C×H and 0.24 in S×A). CV and independent validation methods were used to test the accuracy of genomic predictions. CV was used to test accuracies within a population and independent validation was used to test accuracies across the populations. Independent validation was performed using the marker effects from the C×H training population to estimate GEBVs of the S×A testing population.

### Prediction accuracy of TYM

#### Cross-validation:

We used genotype data of 1975 SNPs in the C×H population and 1483 SNPs in the S×A population for testing within-population prediction accuracies. Missing genotypes were estimated with the mean imputation method implemented in the “rrBLUP” package and the proximity matrix method implemented in the “randomForests” package. We observed similar accuracies with both methods. To be able to compare within-population accuracies (CV) with across-population accuracies (independent validation), we used 808 markers that were common to both populations for testing accuracies in each population. Therefore, in each population CV accuracies were tested with full as well as reduced set of 808 markers.

**Figure 1:**
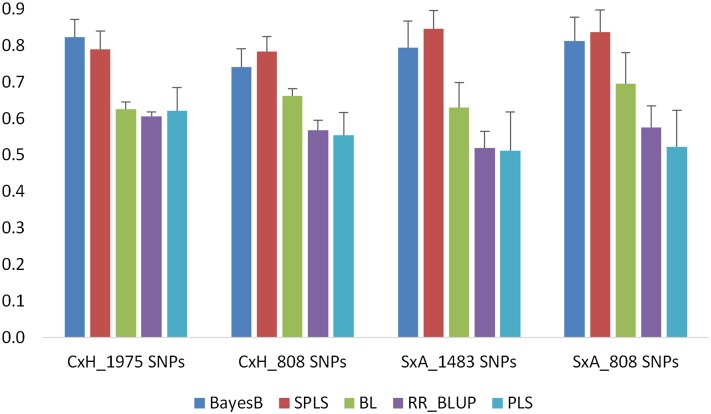
Prediction accuracies obtained for TYM in C×H and S×A populations using 10-fold CV. Analyses were conducted with population-specific SNPs (1975 SNPs for C×H and 1483 SNPs for S×A) as well as 808 SNPs that are common to both the populations. Error bars are standard errors of mean from 10 repeats.

For TYM, prediction accuracies ranging from 0.61 to 0.82 were observed in the C×H population using the 1975 SNPs in CV. Slightly lower accuracies (ranging between 0.55 and 0.78) were observed with the common 808 markers compared to a full set of markers (Figure 1). In the S×A population, prediction accuracies ranged between 0.51 and 0.84 with all 1483 SNPs. Accuracies with the common 808 markers are similar to those with full set of markers ranging between 0.52 and 0.84 (Figure 1). In all these analyses, BayesB and SPLS models yielded the highest accuracies, followed by BL, whereas RR-BLUP and PLS models yielded the lowest accuracies.

#### Independent validation:

In independent validation, marker effects of TYM estimated in the C×H population were used to develop the prediction model that is then used to predict the GEBVs in the S×A population. We used C×H population as a training population because this population was used in our earlier QTL studies to identify the QTL linked to the two traits used in this study. The S×A population was used as a testing population because it is an independent population not used in our previous QTL studies and the parents of this population are different from C×H population. In independent validation, prediction accuracies were tested with the 808 common markers.

Prediction accuracies of TYM in the S×A population were generally lower than CV and ranged between 0.32 and 0.70 (Table 1). Similar to the results from CV, higher accuracies were observed with BayesB (0.70) and SPLS (0.66), followed by BL (0.59), whereas RR-BLUP (0.36) and PLS (0.32) produced the lowest accuracies. Next, we tested the accuracies of prediction models using only the 42 markers from chromosome 5A, which harbored the main TYM QTL observed in our previous study. Higher accuracies were observed with 42 QTL-linked SNPs compared to the 808 common markers (Table 1). Accuracies among all the five models were similar (ranging between 0.68 and 0.71). However, the increase in accuracy using the QTL-linked markers compared to 808 common markers was large for PLS (0.69 *vs.* 0.32) and RR-BLUP (0.68 *vs.* 0.36) compared to BL (0.70 *vs.* 0.59), SPLS (0.70 *vs.* 0.66), and BayesB (0.71 *vs.* 0.70). BayesB and SPLS had the least improvement in accuracy, indicating the ability of these models to accurately select the large effect markers from 808 markers when making prediction. To test the importance of the QTL-linked SNPs in predictions, we analyzed the accuracies by excluding the 42 QTL-linked markers from the 808 common markers. Prediction accuracies were very low and close to zero for all models when the QTL-linked markers were excluded from the models (Table 1).

**Table 1:** Prediction accuracies of TYM and SGN in the S×A population using the models developed in the C×H population

Trait	BayesB	SPLS	BL	RR-BLUP	PLS
TYM (808 SNPs)*^a^*	0.70	0.66	0.59	0.36	0.32
TYM (42 SNPs)*^b^*	0.71	0.70	0.70	0.68	0.69
TYM (766 SNPs)*^c^*	−0.03	0.06	−0.07	−0.06	−0.03
SGNC (808 SNPs)*^a^*	0.22	0.24	0.16	0.13	0.12
SGNC (42 SNPs)*^b^*	0.21	0.21	0.21	0.20	0.19
SGNC (766 SNPs)^c^	0.001	0.03	0.000	0.003	0.000
SGNO (808 SNPs)*^a^*	0.06	0.10	0.05	0.07	0.11
SGNO (42 SNPs)*^b^*	0.26	0.23	0.28	0.31	0.30
SGNO (766 SNPs)*^c^*	0.00	0.06	−0.04	0.01	0.00

TYM, time to young microspore; SGNC, spike grain number under control conditions; SGNO, spike grain number under osmotic conditions; SPLS, sparse partial least squares; BL, Bayesian LASSO; RR-BLUP, Ridge regression best linear unbiased prediction; PLS, partial least squares.

a 808 SNPs are common to both C×H and S×A biparental populations.

b 42 SNPs from chromosome 5A where QTL were identified for TYM and SGNO in a separate study.

c 42 QTL associated SNPs were excluded from 808 common SNPs.

### Prediction accuracy of SGN

#### Cross-validation—SGNC:

Ten-fold CV was used to test the prediction accuracies for SGN under control (SGNC) and osmotic (SGNO) conditions in each population separately.

**Figure 2:**
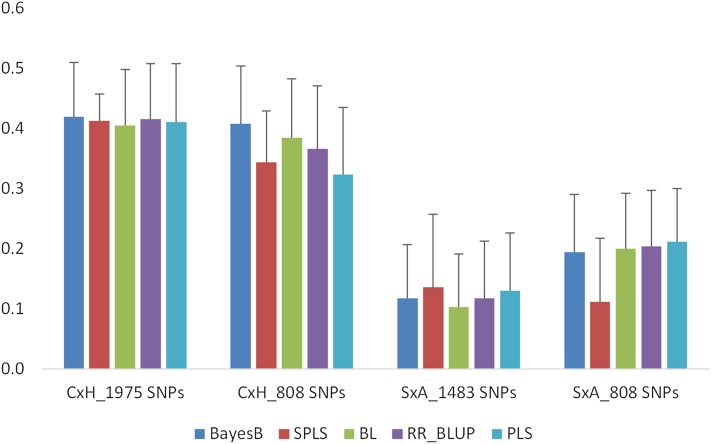
Prediction accuracies for SGNC in C×H and S×A populations using 10-fold CV. Analyses were conducted with both population-specific SNPs (1975 SNPs in C×H and 1483 SNPs in S×A) and 808 SNPs that are common to both the populations. Error bars are standard errors of mean from 10 repeats.

Prediction accuracies were lower with high error rates in the S×A population compared to the C×H population under control conditions (Figure 2). In the C×H population, accuracies ranged between 0.40 and 0.42, whereas in the S×A population they are between 0.10 and 0.14 using the full set of markers. When only the 808 common SNPs were used in prediction models, accuracies were either similar or slightly lower than those with all markers in the C×H population (ranging between 0.32 and 0.41). However, in the S×A population accuracies with the 808 common markers are higher than those using all markers (ranging between 0.11 and 0.21) for all models except for SPLS. Prediction accuracies with either a full set or a reduced set of markers are similar among all the five models in both populations except for SPLS. Prediction accuracy for SPLS is lower compared to other models with a reduced set of markers in the S×A population.

#### Cross-validation—SGNO:

**Figure 3:**
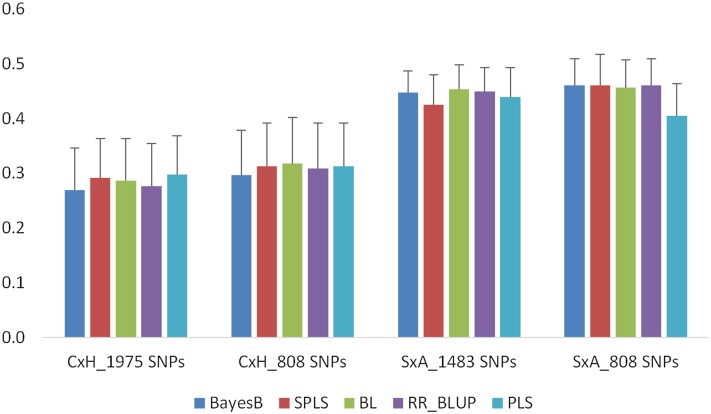
Prediction accuracies for SGNO in C×H and S×A populations using 10-fold CV. Analyses were conducted with both population-specific SNPs (1975 SNPs in C×H and 1483 SNPs in S×A) and 808 SNPs that are common to both populations. Error bars are standard errors of mean from 10 repeats.

In stress treatment, the prediction accuracies in contrast to the control treatment are lower in the C×H population than those in the S×A population with both a full set of markers and common markers (Figure 3). In the C×H population accuracies ranged between 0.27 and 0.30 with a full set of markers (1975 SNPs), whereas in the S×A population they ranged between 0.43 and 0.45 with a full set of markers (1483 SNPs). Accuracies with the common 808 markers are similar to a full set of markers in both populations. In the C×H population, accuracies ranged between 0.30 and 0.32, whereas in the S×A population the accuracies ranged between 0.41 and 0.46. Similar to the control treatment, the accuracies among all five models are very similar in stress treatment (Figure 3).

#### Independent validation—SGNC:

In independent validation, marker effects of SGN in the C×H population were used to develop the prediction model for control (SGNC) and osmotic (SGNO) treatments separately with the 808 common markers. This model was then used to predict GEBVs in the S×A population. Under control conditions, SPLS and BayesB had the highest accuracies (0.24 and 0.22, respectively), followed by the BL (0.16), whereas PLS and RR-BLUP had the lowest accuracies (0.12 and 0.13, respectively; Table 1). We then compared the accuracies of independent validation (across population) with CV (within population) obtained with the 808 common markers. Accuracies with independent validation were lower than those with CV in the S×A population for all models except for SPLS and BayesB (Table 1 and Figure 2). For SPLS, the accuracy has increased from 0.11 in CV to 0.24 in independent validation, and for BayesB there is a slight increase in the accuracy with independent validation compared to CV (Table 1 and Figure 2) in the S×A population. Similar to TYM, we observed a QTL associated with SGNO on chromosome 5A in our previous QTL studies. To test if the 42 markers from chromosome 5A that are associated with SGN under osmotic conditions can predict SGN under control conditions, we used only these 42 markers in the prediction models. Prediction accuracies have slightly increased for BL, RR-BLUP, and PLS but decreased for SPLS and BayesB with QTL-linked markers compared to all 808 common markers (Table 1). However, when the QTL-linked markers were excluded from the 808 common marker set, accuracies were close to zero for all five models (Table 1).

#### Independent validation—SGNO:

In contrast to the control treatment, accuracies in osmotic treatment were very low for all five models using the 808 common markers (Table 1). The accuracies ranged from 0.11 (PLS) to 0.05 (BL). However, using only the QTL-linked markers the accuracies have increased for all five models (Table 1). Accuracies with QTL-linked markers ranged from 0.23 (SPLS) to 0.31 (RR-BLUP). These accuracies are higher than those observed under control conditions (Table 1). Similar to control conditions, accuracies obtained after excluding the QTL-linked markers were close to zero for all models.

## Discussion

### Prediction accuracies among different models

In this study, we used different prediction models to compare the accuracies of flowering time and grain number traits in wheat. BayesB and SPLS outperformed all other models, followed by BL in predicting TYM (Figure 1). The performance of all five models is similar in predicting SGNC and SGNO (Figure 2 and Figure 3) with CV. The differences in prediction performance of different models reflect the underlying genetic architecture of the traits. TYM is a trait influenced by a few loci with major effects. In a recent QTL study, we identified a major QTL on chromosome 5A explaining 72% of phenotypic variation in TYM. SPLS, BayesB, and BL are regarded as variable selection models (Pérez *et al.* 2010). Because of the different underlying assumptions of these models compared to others, BayesB and BL identify a subset of markers with large effects to make predictions that increase the prediction accuracy of the traits, especially those controlled by a few large QTL. SPLS combines variable selection and modeling in one step (Le Cao *et al.* 2008). For traits that are influenced by several loci, such as SGN, the performance of all models was similar with CV (Figure 2 and Figure 3). Several studies have shown better performance of BayesB in predicting traits influenced by a few genes of large effect (VanRaden *et al.* 2009; Daetwyler *et al.* 2010; Jannink *et al.* 2010). Studies have also shown that accuracies from GBLUP, which is equivalent to RR-BLUP and BayesB, are similar in predicting quantitative traits (Clark *et al.* 2011). Daetwyler *et al.* (2010) have shown through simulations that BayesB has an advantage over GBLUP when the number of QTL underlying a trait are small, and this advantage is diminished when the number of QTL increased similar to the results observed in this study. However, Riedelsheimer *et al.* (2012) did not find any major differences between BayesB, RR-BLUP, and other models in predicting several traits, including traits with large QTL effects. They attributed this to the high level of LD among the diverse maize lines used in their study. Results from our study, however, show that even when LD is high within a population the performance of BayesB and SPLS is higher than that of other models, especially for traits with large QTL effects.

### Prediction accuracies of TYM

Accuracies from CV are higher than those from independent validation, where the prediction model developed in C×H was used to predict flowering time in S×A. In independent validation, prediction accuracies using the 42 chromosome 5A SNPs from QTL region are higher than those from the 808 common SNPs for all models except BayesB and to some extent for SPLS. For BayesB and SPLS, however, there is no big change in the accuracy using either 42 SNPs from the QTL region or all the 808 common SNPs (Table 1). This indicates that BayesB and SPLS are able to correctly select the subset of markers with a large effect from all the SNPs when predicting the trait. This trend is also seen to a certain extent with BL, which is another variable selection model. With RR-BLUP and PLS, there is more than 80% improvement in accuracies using the linked markers compared to the random markers (Table 1). RR-BLUP assumes equal variance and shrinks all the marker effects to zero, leading to lower accuracy, especially when there are some large-effect QTL present among the markers. Although PLS reduces dimensions, it does not automatically select variables and uses all markers in the model (Chung and Keles 2010).

### Prediction accuracies for SGN

Accuracies for SGN are generally lower compared to TYM, reflecting the lower heritability estimates observed for SGN compared to TYM. This may also reflect the differences in genetic architecture of these traits. Our earlier QTL studies in the C×H population have shown that TYM is influenced by a major QTL with large effects, whereas SGNO is influenced by several QTL with smaller effects (R. Dolferus, X. Ji, S. Thavamanikumar, E. Tanaka, J. Edlington, K. Forrest, G. Rebetzke, M. Hayden and B. Cullis, unpublished data). Of these QTL, one QTL from chromosome 5A has accounted for a significant portion of variation in TYM (71%) and SGN (30%). Accuracies for SGNC are lower in independent validation than CV using the 808 common markers in the S×A population for all models except for SPLS and BayesB (Figure 2; Table 1). For SPLS and BayesB, there was an increase in accuracy with independent validation compared to CV in the S×A population. However, when only the 42 QTL-linked markers from chromosome 5A are used in independent validation, prediction accuracies were higher than those with 808 common SNPs for all models (Figure 2). These results indicate that SPLS, BayesB, and to some extent BL are able to select a subset of markers with large effects when predicting SGNC similar to TYM. For cross-population predictions, prediction models that capture LD between markers and QTL are more important than those capturing genetic relationships.

For SGNO, accuracies were substantially lower with independent validation compared to CV with 808 markers in the S×A population (Table 1 and Figure 3). This could be due to these two populations responding to osmotic stress differently because of the differences in the genetic background, leading to differences in marker effects between the two populations. Similar to control treatment, accuracies using 42 SNPs from chromosome 5A are higher than those from 808 markers with independent validation (Table 1). However, none of the models were able to identify the large-effect markers correctly from 808 markers, leading to lower accuracies with 808 markers compared to 42 QTL-linked markers. The improvement in accuracies when using QTL-linked markers is higher for osmotic treatment compared to control conditions (Table 1). This may reflect the method used to detect the QTL in our previous study. We used contrast BLUPs (difference between control and stress treatment) estimated with a mixed model analysis for detecting QTL for SGNO. Therefore, these QTL maintain grain number under osmotic conditions.

### Within population *vs.* across population accuracies

There are several studies in wheat where GS was studied using biparental populations (Heffner *et al.* 2011), elite breeding lines (Poland *et al.* 2012; Storlie and Charmet 2013; Crossa *et al.* 2010), and diverse landraces (Daetwyler *et al.* 2014). In all these studies, the prediction accuracy of GS was assessed by using CV methods in which the same population was used for training as well as testing the accuracies. Although CV methods are useful to assess the accuracies in populations of the same genetic background, these accuracies may not be generalized to other populations with different genetic backgrounds. CV methods in general overestimate the potential of genomic prediction (Hofheinz *et al.* 2012). This is reflected by the high accuracies observed in general with CV compared to independent validation in this study.

In addition to CV, we used independent validation to assess the prediction accuracies. In independent validation, prediction models developed in one population (C×H, training population) are applied in a different population (S×A, testing population) to estimate the GEBVs. The training population (C×H) was used in our previous QTL studies to identify QTL linked to the traits studied in this study, whereas the testing population (S×A) is an independent population. The two DH populations used in this study do not have the same parents. Yet, the accuracies observed with independent validation are as good as CV in the S×A population for TYM and SGNC (Table 1, Figure 1, and Figure 2). This is in contrast to the findings of Riedelsheimer *et al.* (2013), who observed zero or negative accuracies when training and validation populations were unrelated. Several other studies have also shown low accuracy when training and testing populations are unrelated (Hickey *et al.* 2014; Hayes *et al.* 2009a; Asoro *et al.* 2011). Differences between our study and other studies may reflect the difference in the factors that contribute to prediction accuracy of GS models. Within a biparental population, the accuracy of GS is determined by the relationships captured by the markers and markers that are linked to QTL (Riedelsheimer *et al.* 2013). Different models use these two types of information differently. RR-BLUP mainly captures the genetic relationships, whereas BayesB uses LD between markers and QTL in making predictions (Habier *et al.* 2007). Accuracies of the models using genetic relationships decay over generations. However, accuracies of the models using LD between markers and QTL persist for several generations (Habier *et al.* 2007). In the populations used in this study, it seems that the accuracy is influenced more by LD between the markers and QTL than the genetic relationships. In CV within a population, the performance of all the models is similar, especially for SGN (Figure 2 and Figure 3), suggesting accuracies of the models that predominantly use the genetic relationships (RR-BLUP) and those that use marker–trait associations (BayesB and SPLS) are similar. In independent validation, with unrelated training and test populations, accuracy of BayesB and SPLS is higher than all other models. BayesB and SPLS capture the LD between markers and QTL, leading to higher accuracy, whereas RR-BLUP, which captures genetic relationships, performed poorly when the training and testing populations are unrelated. This is further evidenced by the zero or negative accuracies (Table 1) observed for both TYM and SGN when the markers from the 5A chromosome linked to the QTL are excluded from the prediction model in independent validation.

Our earlier QTL study indicated that chromosome 5A harbors the strongest QTL for both TYM and SGN. Results from this study validate these results in an independent population (S×A population). Higher accuracies observed in the S×A population with the 42 QTL-linked SNPs compared to the random SNPs indicate that the QTL observed in our earlier study are mainly due to LD between markers and traits. In this study, we have observed higher accuracies with QTL-linked markers compared to the random markers, especially in independent validation. Improvement in prediction accuracies by using “prior information” from association and QTL studies has been suggested previously (Thavamanikumar *et al.* 2013; Zhang *et al.* 2014). Several studies have shown higher accuracies with QTL-linked markers compared to random markers (Zhang *et al.* 2014; Westbrook *et al.* 2013). In a recent study in rice, it was suggested to use markers detected with genome-wide association studies in GS rather than random markers to increase the efficiency and accuracy of GS (Spindel *et al.* 2015). Similarly, in a recent study of wheat, Zhao *et al.* (2014) observed higher accuracies for heading time with marker-assisted selection using functional markers compared to genomic selection with random markers. They developed a method in which functional markers were given more weightage compared to random markers, which increased the prediction accuracy. The importance of including markers identified from QTL and association studies in prediction models is demonstrated in this study by the low accuracy observed when the QTL-linked markers were excluded from the models.

Although a few studies have shown higher accuracies with QTL-linked markers, especially for traits influenced by a few loci with large effects (Zhao *et al.* 2014 and Spindel *et al.* 2015), several studies have shown that the prediction accuracies with marker-assisted selection or marker-assisted recurrent selection in which only a few QTL-linked markers are used for prediction (Bernardo 2008) were lower than those with GS (Heffner *et al.* 2011; Guo *et al.* 2012; Lorenzana and Bernardo 2009). These findings are in contrast to the results from this study. We observed higher accuracies with a few QTL-linked markers compared to several random markers for both TYM and SGN in this study (Table 1). This may be due to the differences in relatedness between training and testing populations in our study compared to other studies. In most of the previous studies, prediction accuracies of marker-assisted selection and GS were compared using CV with related training and testing populations. In this study, however, accuracies were estimated with unrelated training and testing populations. When training and testing populations are related, capturing relationships with GS may lead to higher accuracy than using only the significant markers in marker-assisted selection. However, when training and testing populations are unrelated, accuracy of marker-assisted selection is higher because there would be fewer relationships to be captured with GS and the nonsignificant markers may add to the noise, thus decreasing the accuracy of the GS. Including QTL-linked markers in the panel of GS markers therefore may increase the prediction accuracies, especially when the training and testing populations are unrelated.

## Conclusions

In this study, we compared the accuracies of flowering time and grain number traits in wheat using different GS models. BayesB and SPLS outperformed other models in predicting time to young microspore, whereas their performance is similar to other models in predicting grain number per spike when training and testing populations are related. However, when the training and testing populations are unrelated, BayesB and SPLS are effective in capturing LD between markers and QTL, leading to higher accuracy for both simpler and complex traits compared to other models. Results from this study indicate that the accuracy of the GS models can be increased by using markers identified from linkage and association studies.
